# Cleavage of the urokinase receptor (uPAR) on oral cancer cells: regulation by transforming growth factor – β1 (TGF-β1) and potential effects on migration and invasion

**DOI:** 10.1186/s12885-017-3349-7

**Published:** 2017-05-19

**Authors:** Synnove Norvoll Magnussen, Elin Hadler-Olsen, Daniela Elena Costea, Eli Berg, Cristiane Cavalcanti Jacobsen, Bente Mortensen, Tuula Salo, Inigo Martinez-Zubiaurre, Jan-Olof Winberg, Lars Uhlin-Hansen, Gunbjorg Svineng

**Affiliations:** 1 Department of Medical Biology, Faculty of Health Sciences, UiT – The Arctic University of Norway, N-9037 Tromsø, Norway; 2 Diagnostic Clinic – Clinical Pathology, University Hospital of North Norway, Tromsø, Norway; 3 Gade Laboratory for Pathology, Department of Clinical Medicine, Faculty of Medicine and Dentistry, University of Bergen, Bergen, Norway; 4 Department of Pathology, Haukeland University Hospital, Bergen, Norway; 5 Cancer and Translational Research Medicine Unit, University of Oulu, Oulu, Finland; 6 Medical Research Center, Oulu University Hospital, Oulu, Finland; 7 Oral and Maxillofacial diseases, Clinicum, University of Helsinki, Helsinki, Finland; 8 Helsinki University Hospital Helsinki, Helsinki, Finland; 9 Department of Oral Diagnosis, Oral Pathology Division, Piracicaba Dental School, University of Campinas, Piracicaba, São Paulo SP-13414-903 Brazil; 10 Department of Clinical Medicine, Faculty of Health Sciences, UiT – The Arctic University of Norway, Tromsø, Norway

**Keywords:** Urokinase plasminogen activator receptor (uPAR), Urokinase receptor, Transforming growth factor-beta1 (TGF-β1), Plasminogen, Plasmin, Cancer, Cell migration, Urokinase, Invasion

## Abstract

**Background:**

Urokinase plasminogen activator (uPA) receptor (uPAR) is up-regulated at the invasive tumour front of human oral squamous cell carcinoma (OSCC), indicating a role for uPAR in tumour progression. We previously observed elevated expression of uPAR at the tumour-stroma interface in a mouse model for OSCC, which was associated with increased proteolytic activity. The tumour microenvironment regulated uPAR expression, as well as its glycosylation and cleavage. Both full-length- and cleaved uPAR (uPAR (II-III)) are involved in highly regulated processes such as cell signalling, proliferation, migration, stem cell mobilization and invasion. The aim of the current study was to analyse tumour associated factors and their effect on uPAR cleavage, and the potential implications for cell proliferation, migration and invasion.

**Methods:**

Mouse uPAR was stably overexpressed in the mouse OSCC cell line AT84. The ratio of full-length versus cleaved uPAR as analysed by Western blotting and its regulation was assessed by addition of different protease inhibitors and transforming growth factor - β1 (TGF-β1). The role of uPAR cleavage in cell proliferation and migration was analysed using real-time cell analysis and invasion was assessed using the myoma invasion model.

**Results:**

We found that when uPAR was overexpressed a proportion of the receptor was cleaved, thus the cells presented both full-length uPAR and uPAR (II-III). Cleavage was mainly performed by serine proteases and urokinase plasminogen activator (uPA) in particular. When the OSCC cells were stimulated with TGF-β1, the production of the uPA inhibitor PAI-1 was increased, resulting in a reduction of uPAR cleavage. By inhibiting cleavage of uPAR, cell migration was reduced, and by inhibiting uPA activity, invasion was reduced. We could also show that medium containing soluble uPAR (suPAR), and cleaved soluble uPAR (suPAR (II-III)), induced migration in OSCC cells with low endogenous levels of uPAR.

**Conclusions:**

These results show that soluble factors in the tumour microenvironment, such as TGF-β1, PAI-1 and uPA, can influence the ratio of full length and uPAR (II-III) and thereby potentially effect cell migration and invasion. Resolving how uPAR cleavage is controlled is therefore vital for understanding how OSCC progresses and potentially provides new targets for therapy.

## Background

Oral squamous cell carcinoma (OSCC) is characterized by aggressive behaviour, including local invasion and metastasis to lymph nodes [1, 2]. Expression of the urokinase plasminogen activator (uPA) receptor (uPAR) has been reported to be elevated at the tumour-stroma border of many cancer types [3–5], including OSCC [6, 7], indicating a role of uPAR in cancer invasion. uPAR is involved in binding and activation of the protease uPA. Once activated, uPA can proteolytically cleave plasminogen, producing the active broad spectrum serine protease plasmin needed in normal physiological processes such as wound healing [8]. In a feedback-loop fashion, plasmin activates uPA, but also several matrix metalloproteases (MMPs) and growth factors. Plasmin may be inhibited by α2-antiplasmin, α2-macroglobulin, thrombin activatable fibrinolysis inhibitor (TAFI) and protease nexin-1 (PN-1), while uPA is inhibited mainly by plasminogen activator inhibitor-1 (PAI-1) and −2 (PAI-2) [8, 9]. Higher levels of uPA, uPAR and PAI-1 correspond to more aggressive disease for prostate-, cervical-, liver- and oral cancer [8], where uPA and PAI-1 have been validated as strong and independent prognostic factors for poor survival in primary breast cancer [10]. We recently reported that low expression of uPAR and PAI-1, was correlated with longer disease specific survival in early stage OSCC [11]. Furthermore, one of the key regulators of PAI-1 expression, transforming growth factor β1 (TGF-β1), is increased in pre-malignant oral leukoplakia and in OSCC compared to normal oral mucosa [12, 13].

uPAR is GPI-anchored to the cell membrane and hence locates proteolytic activity to the cell surface, which is needed for the invasive process, as seen during wound healing and cancer invasion [14, 15]. Both human and murine uPAR consists of a single polypeptide chain that forms a 3D structure consisting of three homologous domains, known as domains I, II and III, where the GPI-anchor is attached to the third domain. These three domains create an internal cavity where pro-uPA can bind via its amino terminal fragment (ATF) and become activated [16–18]. Once activated, uPA, along with a spectrum of other proteases, including plasmin, chymotrypsin, cathepsin G, elastase and MMP’s [19–22], can cleave uPAR creating a shorter protein containing only domains II and III, termed uPAR (II-III) [23]. uPA-induced cleavage renders uPAR (II-III) on the cell surface, now unable to bind uPA [24, 25]. Even though uPAR lacks an intracellular domain, the receptor is involved in cell signalling, mainly through the interaction with neighbouring receptors [26], where full-length uPAR and uPAR (II-III) engage different signalling pathways [27]. uPAR can also be shed from the cell surface, producing soluble variants of uPAR, namely full-length soluble uPAR (suPAR) or cleaved soluble uPAR; suPAR (II-III) [28] and suPAR (I). These uPAR fragments are correlated with survival in many cancer types [29–32] and several studies indicate that uPAR (II-III) and suPAR (II-III) are involved in highly regulated processes such as cell signalling [28] and stem cell mobilization [33, 34]. Today, little is known about how uPAR cleavage is regulated and the consequences this has on cancer progression. We recently showed that the tumour microenvironment (TME), mainly through soluble factors, readily up-regulated the expression and cleavage of uPAR in mouse OSCC cells [35]. The TME consists of different cell types such as immune cells, endothelial cells and fibroblasts, as well as structural matrix proteins, insoluble and soluble factors such as cytokines, chemokines and growth factors, including TGF-β1 [36]. TGF-β1 is a fundamental regulatory molecule of the tumour microenvironment and may be expressed by tumour-associated macrophages (TAMs), cancer-associated fibroblasts (CAFs) and cancer cells [37–39].

The aim of the current study was to analyse the regulation of uPAR expression and cleavage by uPA and TGF-β1, and the potential implications on migration and invasion of the mouse OSCC cells AT84. We found that TGF-β1 reduced uPAR cleavage through up-regulation of PAI-1 expression, increasing the amount of full-length uPAR present on the AT84 OSCC cells. Both cell surface associated- and shed uPAR (suPAR and suPAR (II-III)) were found to regulate cell migration and invasion. Inhibiting uPA activity, and thus uPAR cleavage, with the uPA-specific inhibitor BC11 hydrobromide, resulted in reduced migration and invasion. In conclusion, these results demonstrate that the ratio of full-length versus cleaved uPAR can be regulated by TGF-β1, PAI-1 and uPA which may subsequently affect cell migration and invasion.

## Methods

### Materials

Bovine serum albumin (BSA) (A9647, lot: SLBC9771V), aprotinin from bovine lung (A3428, lot: 060M70081V), NaHCO_3_-buffered RPMI-1640 with L-glutamine (R8758), Dulbecco’s Modified Eagle Medium (DMEM; D5796), foetal bovine serum (FBS) (F7524, lot: 011 M3398), puromycin dihydrochloride (P9620), DL-Dithiothreitol (DTT) (43,815, lot: BCBK8939V), SIGMAFAST™ Protease Inhibitor Cocktail (S8830-20TAB, lot: SLBG7024V), penicillin and streptomycin mix (P4333), the TGF-β1 inhibitor SB431542 (S4317, lot: 104M4747V) were purchased from Sigma Aldrich (St. Louis, MO, USA). The QIAshredder kit (79654), RNeasy kit (74134), QuantiTect Reverse Transcription Kit (205313) and primers (uPAR: QT00102984, uPA: QT00103159, Plasminogen: QT01053332, βactin: QT00095242, and TRFC: QT00122745) were purchased from Qiagen (Hilden, Germany). The Faststart Essential DNA Green Master (06402712001) was purchased from Roche Diagnostics (Indianapolis, IN). The Direct Detect system (DDAC00010-GR, lot: 39,591–1-9), PVDF membranes (IPVH00010), Re-Blot Plus Mild Solution (2502) were all from EMD Millipore Corp. (Billerica, MA). BC11 hydrobromide (4372, Batch no. 1A/117980) was purchased from Tocris Bioscience (Ellisville, MO) and TGF-β1 (100-B-001, lot: A5013041) from RD Systems (Minneapolis, MN). Recombinant murine PAI-1 (rmPAI-1) (528,213, Lot: D00138824) was purchased from Calbiochem, EMD Chemicals Inc. (San Diego, CA). Purified mouse high molecular weight (HMW)-uPA (MUPA) and mouse plasmin (MPLM) were from Molecular Innovations (Novi, MI). Plasminogen (plg) from human plasma (528,175, Lot: D00156550) was purchased from Merck KGaA (Darmstadt, Germany). The Gateway® cloning system and the Bis-Tris SDS-gels were bought from Invitrogen (Carlsbad, CA). EDTA (20,302.293, lot: 09 K26007) was purchased from VWR International (Leuven, Belguim). Opti-MEM (31985–047) was purchased from Gibco (Paisley, UK). The PNGase F kit (P0704S) was from New England BioLabs (Beverly, MA). Biotinylated protein ladder (7727, lot: 21) was from Cell Signaling Technology (Danvers, MA). Western blotting Luminol Reagent (sc-2048) was from Santa Cruz Biotechnology Inc. (Frederick, MD). The polink-2 Plus HRP Detection kit for goat primary antibody was from GBI Labs (Mukilteo, WA). The following machines and software were purchased as follows: SPSS Statistics 19 for Windows from SPSS Corp. (Chicago, Il), CDF320 camera, DCF425 camera, IM50 software, Leica Application Suite (LAS version 3.7.0) from Leica Microsystems (Heerburg, Switzerland), SigmaPlot from Systat Software Inc. (London, UK) and Olympus DP software, Soft 5.0 (Olympus Corporation, Tokyo, Japan). The LightCycler 96 and the xCELLigence system were from Roche Diagnostics (Mannheim, Germany), LAS-3000 imaging system was from Fujifilm (Tokyo, Japan). The NanoDrop spectrophotometer was from Thermo Scientific (Wilmington, DE), the Experion automated electrophoresis system was from Bio-Rad Laboratories (Hercules, CA). The BD FACSAria was from BD Biosciences (San Jose, CA), and FlowJo software (version 7.6.5) was from Tree star Inc. (Ashland, OR).

### Antibodies

Antigen affinity-purified polyclonal goat anti-mouse uPAR antibody (AF534, lot no: DCL03112081, DCL0311021) was from R&D Systems (Minneapolis, MN). We have previously demonstrated the antibody specificity in IHC [35]. For Western blotting a dilution 1:1000 or 1:500 was used. To demonstrate the specificity of AF534 in Western blots, a sheep anti-mouse uPAR antibody (CSI19991A, lot no: 2,209,001) from Cell Sciences (Canton, MA) was used in a Western blot at 1:2500 dilution and similar results were obtained (results not shown). In flow cytometer analysis, AF534 was used in 1:100 dilution, and for immunohistochemistry (IHC) a 1:200 dilution for 1 h at room temperature. For flow cytometry, the Alexa Fluor 488 donkey anti-goat antibody (A11055) from Invitrogen (Carlsbad, CA) was used at 1:500. For Western blotting, HRP-conjugated anti goat/sheep (A9452) was used at 1:100,000, and HRP-conjugated anti-β-actin (A3854) at 1:25,000 (Sigma Aldrich, St. Louis, MO). The polyclonal rabbit anti-murine PAI-1 antibody was used for neutralizing murine PAI-1 (50 μg/ml) and for Western blotting at 1:2500 (Ab28207, lot no: 1,060,006) was from Abcam Inc. (Cambridge, MA). Monoclonal rabbit anti-low density LPR (LRP1)(EPR3724, lot no GR47571–2) was diluted 1:2500 (ab92544, Abcam Inc., Cambridge, MA) and both were detected using HRP-conjugated anti-rabbit (4050–05, Southern Biotech, Birmingham, AL). To enable detection of the biotinylated protein ladder, an anti-biotin HRP-linked antibody was used at 1:1000 dilution (7075P5, lot: 30, Cell Signaling Technology, Danvers, MA).

### Cloning and expression of uPAR in cultured AT84 cells

Cloning of uPAR in AT84 cells has previously been described [35], but is summarized in brief. The *Plaur* gene was cloned from the murine macrophage cell line J774 into the mouse cell line AT84 using the Gateway® cloning system. Overexpression of uPAR was achieved through stable transfection of pDest/TO/PGK-puro/uPAR and a mixed population was obtained through puromycin treatment. Using Fluorescence-activated cell sorting (FACS), 11.000 cells expressing high levels of uPAR were sorted for further culturing and denoted AT84-uPAR (see flow cytometry below). Control cells containing only the empty vector, pDest/TO/PGK-puro, were denoted AT84-EV cells. Cell images were recorded using a Leica camera and the IM50 software.

### Cell lines

The mouse tongue SCC cell line AT84, originally isolated from a C3H mouse [40], was kindly provided by Professor Shillitoe, Upstate Medical University, Syracuse, NY [41]. All cells were cultured at 37 °C, 5% CO_2_ in a humid environment. AT84 cells were maintained in RPMI, supplemented with 10% FBS. For AT84 cells overexpressing uPAR, the culture medium was supplemented with 5 μg/ml puromycin.

### Conditioned medium

Eight ml serum free medium (SFM; RPMI-1640) was added to AT84-EV and AT84-uPAR cells at 60–70% confluency in 75 cm^2^ culture flasks. The medium was conditioned for 48 h. When analysing for suPAR, the conditioned medium from the AT84-EV and the AT84-uPAR cells was concentrated from 2 ml to an equal final volume (specified in the figure legend) using the Vivaspin 500, membrane 10,000 MWPO PES. Conditioned medium containing the soluble factors from the tumour microenvironment (TMEM) of the neoplastic leiomyoma tissue was harvested as previously described [35].

### Flow cytometry

Cells were seeded in medium containing 10% FBS and incubated for 24 h, whereupon the medium was exchanged for SFM and the cells incubated for another 24 h. Cells were detached with 1 mM EDTA and washed once in RPMI ^w^/10% FBS. All subsequent washing steps were performed with Opti-MEM containing 1% BSA, and blocking was done with Opti-MEM ^w^/5% BSA. Non-permeablized cells were labelled using the 1:100 goat polyclonal anti-murine uPAR antibody and 1:1000 Alexa Fluor 488 donkey anti-goat secondary antibody in Opti-MEM ^w^/1% BSA. Cells were subsequently analysed and sorted using a BD FACSAria. For each sample, 10,000 cells were gated. Figures were designed using FlowJo.

### Induction and inhibition of uPAR cleavage

Cells were detached using trypsin (0.25% in PBS with 0.05% Na_2_EDTA), counted and equal cell numbers were seeded in serum-containing media and incubated for 24 h. Cells were then treated in an assay specific manner. Culture medium was exchanged for either SFM or culture medium containing 10% FBS (FBSM). Aprotinin (1.6 mM dissolved in water), BC11 hydrobromide (100 mM dissolved in DMSO. Previously specificity tested [35]), TGF-β1 (10 μg/ml dissolved in 4 mM HCl with 1% BSA) and rmPAI-1 (60.5 μM dissolved in 100 mM NaCl, 50 mM sodium acetate, 1 mM EDTA, pH 5.0) were added to the culture medium to a final and optimized concentration specified in the figure legends and indicated in the figures. TGF-β1 signalling was inhibited by adding either 2 ng/ml TGF-β1 and/or 10 μM of the specific TGF-β1 inhibitor SB431542. Conditioned medium and cell lysates were prepared by removing or harvesting the culture media and scraping cells in RIPA buffer (25 mM Tris-HCl, pH 7.6, 150 mM NaCl, 1% Triton-X100, 0.5% sodium deoxycholate, 0.1% SDS) containing 1× SIGMAFAST™ Protease Inhibitor Cocktail.

### Antibody mediated PAI-1 blocking

Cells were seeded as described in the previous section and treated with 2 ng/ml TGF-β1 in FBSM. Cells were simultaneously treated with 50 μg/ml of the anti-PAI-1 antibody (Ab28207) and incubated for 24 h. Controls received either no treatment, or only TGF-β1. Cells were harvested as described in the “Induction and inhibition of uPAR cleavage” section and analysed by Western blotting.

### Deglycosylation by PNGase F treatment

Cell lysates were treated with PNGase F to remove all N-linked glycosylation. The procedure was performed according to the manufacturer’s protocol with some adjustments. In brief, 1× denaturing buffer was added to the cell lysate or conditioned medium and boiled for 10 min. 1× G7 reaction buffer, 1% NP-40 and 0.5 μl PNGase F were added and incubated for 1 h at 37 °C. Samples were then analysed by SDS-PAGE and Western blotting.

### Western blotting

Cells lysates were sonicated, reduced and boiled. Conditioned medium was neither reduced nor boiled. Total protein concentration was assessed using the Direct Detect system. Some samples were deglycosylated using PNGase F, before equal amounts of protein (10–30 μg) were loaded onto NuPAGE Novex 4%–12% Bis-Tris gels, and subjected to non-reducing SDS-PAGE. A biotinylated protein ladder was run on all gels. Proteins were blotted onto PVDF membranes. Blocking was done with 5% non-fat dry milk, or 5% BSA, in Tris-buffered saline (150 mM NaCl, 20 mM Tris, pH 7.4) supplemented with 0.1% Tween 20. Membranes were incubated with the specific primary antibody at 4 °C overnight diluted in blocking buffer. A HRP-conjugated species specific secondary antibody was used to detect the primary antibody. Western blotting Luminol Reagent was used for antibody detection. Equal loading was controlled by re-probing for β-actin. Images were obtained using the LAS-3000 imaging system.

### Reverse transcriptase quantitative PCR (RT-qPCR)

Cultured cells (3.0 × 10^5^ cells) were harvested using 300 μl RTL buffer containing 100 mM DTT. Samples were homogenized using the QIAshredder followed by total RNA extraction using the RNeasy kit. Quantity and purity of the extracted RNA was determined using the NanoDrop. RNA integrity is routinely assessed on random samples using the Experion automated electrophoresis system. mRNA expression levels were analysed using reverse transcription quantitative PCR (RT-qPCR) on a LightCycler 96. cDNA was synthesized from 1 μg total RNA using the QuantiTect Reverse Transcription Kit. Target cDNA, corresponding to 10 ng RNA, was amplified through 40 cycles in a 25 μl qPCR mix containing 1 μl Qiagen primer mix for *uPAR* (QT00102984), *uPA* (QT00103159)*, Plasminogen* (QT01053332), *βactin* (QT00095242) or *TRFC* (QT00122745) and FastStart Essential DNA Green Master mix. A dissociation curve was routinely run at the end of every PCR to verify sample purity, primer specificity and absence of primer dimers. qPCR cycling conditions: Step 1: 95 °C for 10 min. Step 2: 95 °C for 10 s, 60 °C for 10 s and 72 °C for 10 s was repeated 45 times. Step 3 (dissociation curve): 95 °C for 10 s, 65 °C for 60 s and 97 °C for 1 s continuously. Absence of genomic DNA and contaminants was confirmed by performing no reverse transcriptase (NoRT) controls with every round of RNA purification, and non-template controls (NTC) on each primer set, respectively. For each experiment RNA was purified from at least three biological replicates (*N* = 3). Reverse transcription was performed on all biological replicates, and each biological replicate was loaded as two technical replicates per RT-qPCR run. The delta-delta Cq method [42] was used to determine the relative amount of target mRNA in samples normalized against the average expression of the two reference genes *Trfc* and *βactin*. The numbers are presented as fold differences where the lowest value is set to 1.

### Gelatin- and plasminogen-gelatin zymography

Cells were seeded and incubated overnight and washed three times in PBS before the medium was exchanged for SFM, harvested after 24 h and spun down to remove any cells. MMP-2 and MMP-9, as well as uPA and plasminogen levels were assessed by gelatin (gelzym) and combined gelatin-plasminogen zymography (plgzym) respectively, as previously described [43]. When analysing plasminogen activators, a final concentration of 10 μg/ml of plasminogen was added to the gel. As controls, purified mouse HMW-uPA (44 kDa), mouse plasmin (mPLM, 85 kDa), trypsin (24 kDa) and a mixture of both inactive pro-form and active-form of both human MMP-2 monomer (62 and 72 kDa) and MMP-9 monomer (83 and 92 kDa) were used.

### Real-time cell analysis

The xCELLigence system and real-time cell analysis (RTCA) was used to determine the proliferation and migratory capacity of the cells according to the manufacturer’s instructions. Proliferation experiments were performed to determine the optimal cell seeding density (100–300,000 cells), and for both proliferation- and migration studies a total of 30,000 cells were selected for seeding in 100 μl medium. All experiments were performed at least three times (*N* = 3), and two technical replicates were included per experiment.

#### Proliferation

Thirty μl SFM was added to the E-plates and a background reading was performed. Cells were detached using trypsin (0.25% in PBS with 0.05% Na_2_EDTA), counted and seeded. Attaching and proliferating cells were recorded by electrical impedance, measured every 15 min by the electrodes in the bottom of the wells giving the arbitrary “cell index” value, proportional to the cell number. Cell proliferation was assessed on cells seeded in FBSM as a control, or supplemented with BC11 hydrobromide (10 μM), rmPAI-1 (10 nM) or TGF-β1 (2 ng/ml = 167pM) for at least 72 h.

#### Migration

The bottom wells of cell invasion and migration (CIM) plates were loaded with 160 μl FBSM, with or without the presence BC11 hydrobromide (10 μM), rmPAI-1 (10 nM) or TGF-β1 (2 ng/ml = 167pM). For the suPAR chemotaxis experiments the bottom chamber was filled with 160 μl of AT84-EV or AT84-uPAR conditioned medium. A top chamber containing 16 wells, each equipped with an 8 μm pore membrane in the bottom was then mounted onto the bottom chamber. The wells in the top chamber were loaded with 25 μl SFM, and the plate was equilibrated for 1 h at 37 °C, 5% CO_2_, in a humid environment. A background measurement was performed before cells, re-suspended in SFM (with or without inhibitors), were loaded into the wells. Cells were allowed to attach to the well for 15 min at room temperature, before the plate was mounted into the xCELLigence machine. Electrodes located underneath the membrane recorded only the migrating cells for the subsequent 72 h. Electrical impedance was measured every 15 min and translated into the arbitrary “cell index” value, proportional to the cell number.

### Organotypic invasion model

Preparation of the leiomyoma discs and the invasion procedure has previously been described in detail [35]. In brief, discs of freeze-dried benign leiomyoma tumour tissue were rehydrated in SFM overnight. A total of 0.4 × 10^6^ cells suspended in 50 μl SFM were seeded on top of the discs, and three discs were used per cell line (*N* = 3). Cells were allowed to attach and invade the tissue for seven days. Discs were fixed in a zinc-based fixative (ZBF) (36.7 mM ZnCl_2_, 27.3 ZnAc_2_, x2H_2_O and 0.63 mM CaAc_2_ in 0.1 mol/L Tris pH 7.4), dehydrated and paraffin-embedded. Tissue sections of the leiomyoma discs were stained with hematoxylin/eosin (H/E) and a blinded analysis of cell invasion was performed on images using the Olympus DP software, Soft 5.0. A horizontal line was drawn through the uppermost remnants of the leiomyoma tissue in order to set a “basement membrane” level. Invasion depth was determined every 100 μm along the horizontal line as the vertical distance from this line to the limit of invading cells. At least 6 measurements were performed per tissue disc. Leiomyoma tissue without added cells were used as negative controls. Images were recorded using the Leica DCF425 camera and the Leica Application Suite.

### Immunohistochemistry (IHC)

For analysis of uPAR expression, the ZBF fixed leiomyoma discs were IHC stained as previously described [35]. In brief, the primary antibody was diluted in 5% BSA in PBS. For visualization of the uPAR primary antibody, the Polink-2 Plus HRP Detection kit for goat primary antibody was used. The chromogen diaminobenzidine (DAB) was used to visualize the secondary HRP-linked antibody. Sections in which the primary antibody was replaced with 5% BSA were used as negative controls and showed no staining. The specificity of the anti-uPAR antibody has previously been verified for IHC [35].

### Statistical analysis

Data are presented as mean values ± standard deviation (±SD) or ± standard error of mean (±SEM), specified in the figure legends. The differences between groups were assessed using independent-samples T-test. *P*-values <0.05 were accepted as statistically significant. Graphics were made using Excel and Sigma Plot. Statistical analyses were performed using SPSS. Independent replicates (N) for the different data are presented in the figure legends.

## Results

### Active uPA and plasmin are responsible for the majority of uPAR cleavage

**Fig. 1:**
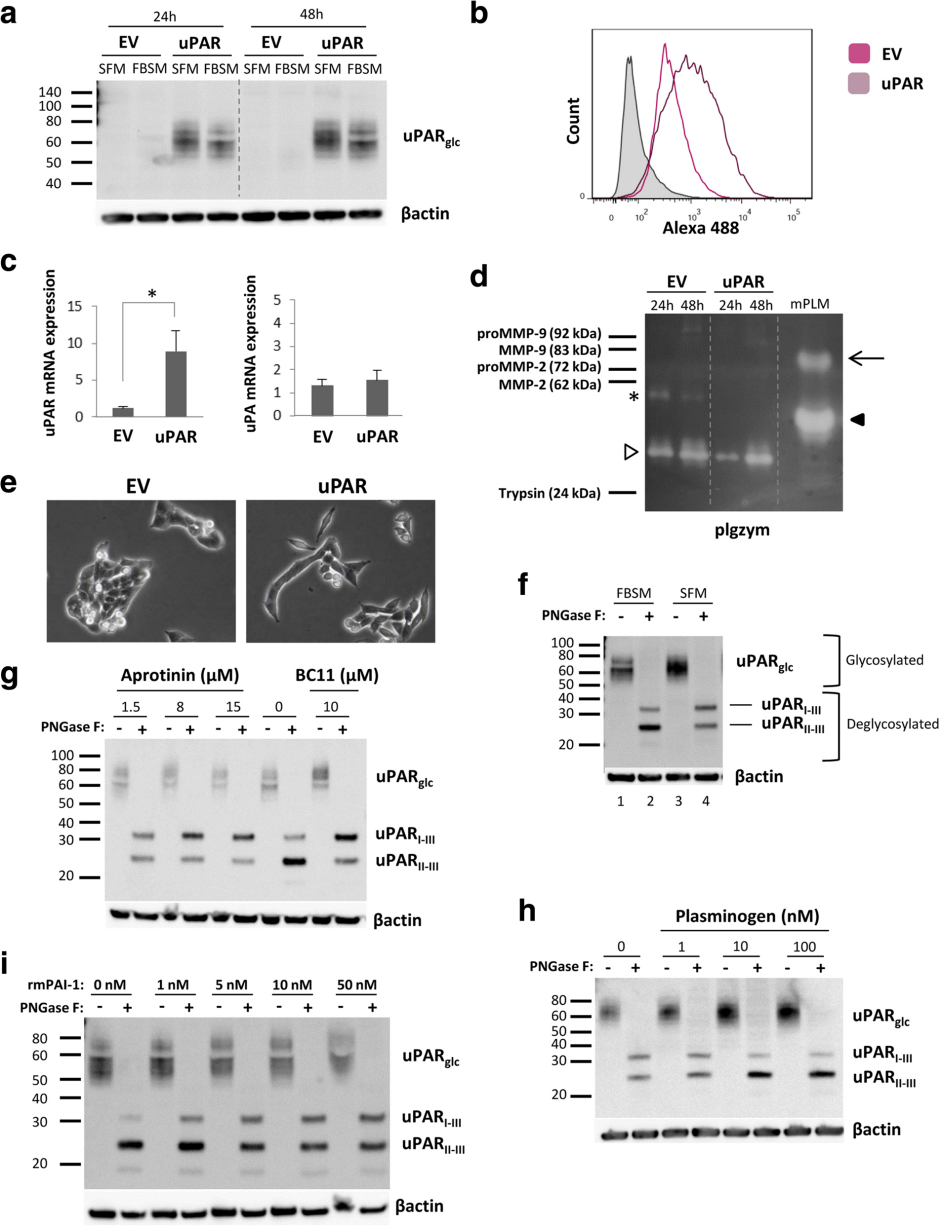
uPAR cleavage is mediated by plasmin and uPA. AT84 cells stably transfected with either empty vector (EV)(AT84-EV) or a vector containing cDNA encoding mouse uPAR (AT84-uPAR) were analysed for uPAR mRNA and protein levels, secreted plasminogen activators and uPAR cleavage by Western blotting of whole cell lysates (A, F-I), flow cytometry (B), RT-qPCR (C) and plasminogen-gelatin zymography (plgzym)(D). **a**. AT84-uPAR (uPAR) or AT84-EV (EV) cells cultured in either serum-free medium (SFM) or medium containing 10% foetal bovine serum (FBSM) for 24- and 48 h. **b**. Non-permeabilized AT84-EV (pink: median fluorescence 371) and AT84-uPAR (purple: median fluorescence 853) cells. Negative control with no primary antibody added (filled curve). **c**. Relative uPAR mRNA (left panel) or uPA mRNA (right panel) expression levels. Error bars represent the standard deviation (+SD) and *N* = 3. Student T-test; * *p* < 0.05. **d**. Conditioned medium from cells cultured for 24- and 48 h in SFM. Positive control: mPLM (mouse plasmin). Active mouse plasmin (arrow), auto-proteolytic fragment of plasmin (black arrowhead), HMW-uPA (white arrowhead), and an unknown plasminogen activator (asterisk). **e**. Images of AT84-EV and AT84-uPAR cells in culture 24 h after seeding (10× magnification). **f-i**. Whole cell lysates of AT84-uPAR cells treated with PNGase F (+) or no PNGase F (−). **f**. Cells cultured in either FBSM or SFM. Glycosylated uPAR (uPAR_glc_), deglycosylated samples gave rise to full length uPAR (uPAR_I-III_) and cleaved uPAR (uPAR_II-III_). **g**. Cells cultured for 24 h in FBSM (0), or FBSM supplemented with either 1.5 μM, 8 μM or 15 μM aprotinin, or 10 μM BC11 hydrobromide. **h**. Cells cultured for 24 h either in SFM without plasminogen (0), or in SFM supplemented with 1 nM, 10 nM or 100 nM plasminogen. **i**: AT84-uPAR cells cultured for 24 h in FBSM supplemented with 1-, 5-, 10- or 50 nM rmPAI-1. Controls (0 nM) received no additives

To follow up our findings from a previous in vivo study [35], the mouse OSCC cell line AT84 was selected also for this study. It enables us the use of a syngeneic mouse model for OSCC in vivo, this being important as there is no species cross-reactivity between human and mouse uPA and uPAR [17]. Furthermore, the AT84 cells express low endogenous levels of uPAR in culture [35]. AT84 cells were stably transfected to overexpress mouse uPAR, and bulk populations of both AT84-uPAR and the empty vector transfected cells (AT84-EV) were generated. The AT84-uPAR cells expressed excess amounts of uPAR protein compared to the AT84-EV cells (Fig. 1a), and the receptor was exposed on the cell surface (Fig. 1b). The AT84-uPAR cells expressed approximately 8-fold more uPAR mRNA compared to the AT84-EV cells as analysed by RT-qPCR (Fig. 1c, left panel). No statistically significant difference in uPA mRNA expression levels could be detected between EV- and uPAR expressing cells (Fig. 1c, right panel). Plasminogen (plg) mRNA levels were also analysed using RT-qPCR, but the expression level was below the limit of detection.

Plasmin, uPA and some MMP’s including MMP-2 and MMP-9 are reported to cleave uPAR [21, 23, 44]. To assess the secreted levels of MMP-2, −9, plasmin and uPA in the conditioned medium of the AT84-EV and AT84-uPAR cells, gelatin zymography (gelzym)(results not shown) and plasminogen-gelatin zymography (plgzym)(Fig. 1d) experiments were performed. Murine plasmin (mPLM) was loaded as a control, and the 85 kDa active plasmin is indicated by an arrow. Plasmin can undergo auto-proteolysis, as seen by the extra band (black arrowhead). Plasmin was not detected in the conditioned medium from the cells (Fig. 1d). As previously reported, both AT84-EV and AT84-uPAR cells expressed detectable levels of a plasminogen activator with the same MW as HMW-uPA (white arrowhead) [35]. The AT84-EV cells did in addition secrete an unknown plasminogen activator (asterisk), which the AT84-uPAR-cells did not secrete. The AT84-EV and AT84-uPAR cells did not secrete any detectable gelatin-degrading enzymes, such as MMP-2 or MMP-9, as analysed by gelzym (results not shown). Taken together, the AT84-EV and -uPAR cells secreted active uPA, but not plasmin, MMP-2 or MMP-9.

Increased expression of uPAR has been reported to alter the morphological appearance of cells in culture and induce epithelial-to-mesenchymal transition (EMT) in breast cancer cells [45–48]. We found that AT84-uPAR cells displayed a more elongated mesenchymal-like morphology compared to the AT84-EV cells (Fig. 1e). However, we did not pursue this further.

Human uPAR can be cleaved between domains I and II, mainly by uPA and plasmin, but also by other proteases [19, 21, 49]. This cleavage releases domain I and leaves a cleaved version of uPAR on the cell surface known as uPAR (II-III). Culturing the AT84-uPAR cells in either serum free medium (SFM) or culture medium containing 10% FBS (FBSM) gave uPAR bands of a slightly different appearance on Western blots (Fig. 1a and Fig. 1f, lane 1 and 3; uPAR_glc_). When the cell lysates were de-glycosylated using PNGase F (Fig. 1f, lane 2 and 4) it was apparent that uPAR was present both in its cleaved (uPAR_II-III_) and full-length version (uPAR_I-III_) as we have also previously reported [35]. As seen in Fig. 1f, the FBSM induced a higher ratio of cleaved uPAR compared to full-length uPAR than SFM. FBS contains many different soluble factors that may induce uPAR cleavage including plasminogen, which may be activated by the uPA produced by the cells. Adding the previously tested [35] specific uPA inhibitor BC11, or the serine protease inhibitor aprotinin, to cells cultured in FBSM resulted in a shift towards more full-length uPAR (Fig. 1g). Accordingly, adding human plasminogen to cells cultured in SFM tilted the ratio towards more cleaved uPAR (Fig. 1h). Addition of recombinant murine PAI-1 (rmPAI-1) also efficiently inhibited uPAR cleavage in FBSM (Fig. 1i), while galardin, a broad-spectrum matrix metalloproteinase (MMP) inhibitor, had no effect on uPAR cleavage in these cells (results not shown). Taken together, these results show that uPA and plasmin are the main regulators of the balance between cleaved and full-length uPAR on the cell surface of AT84 cells. However, additional factors may also play a role since the balance between cleaved and full-length uPAR could not be completely shifted in either direction.

### TGF-β1 reduces uPAR cleavage through induced PAI-1 expression

**Fig. 2:**
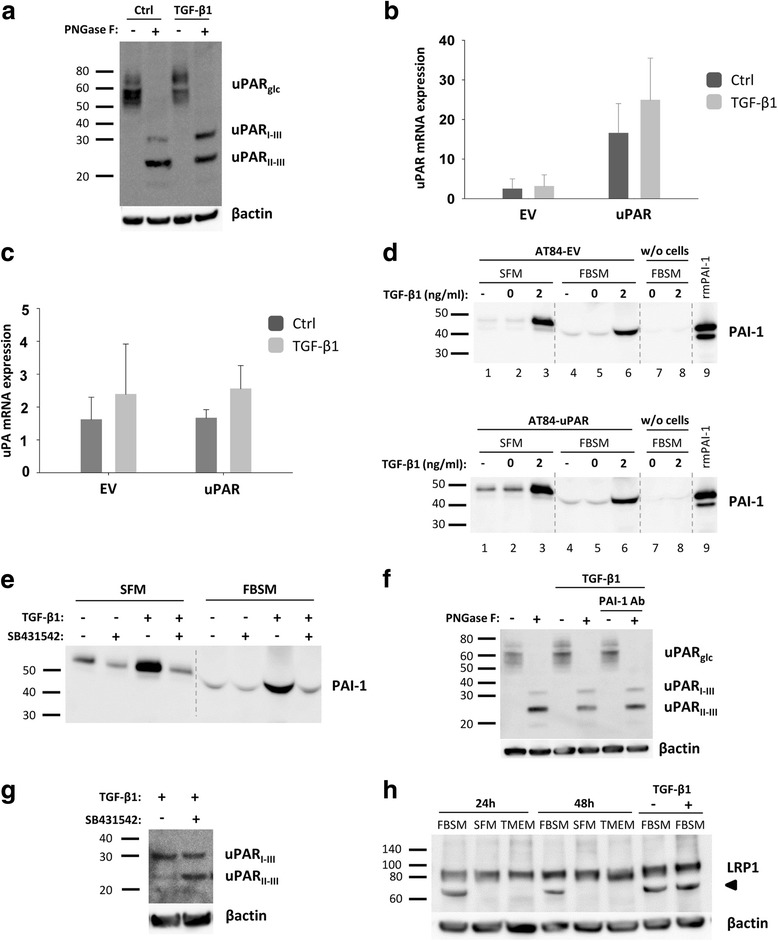
TGF-β1 reduces uPAR cleavage through induced PAI-1 expression. **a**, **f-h**. Western blot analysis of whole cell lysates of cultured and stimulated AT84-uPAR cells. Where indicated, cell lysates were either treated with PNGase F (+) or samples received the same treatment except that PNGase F was omitted (−). Glycosylated uPAR is indicated as uPAR_glc_. **d-e**. Western blot analysis of conditioned medium from equally seeded amounts of AT84-EV and AT84-uPAR cells. **a**. Cells cultured in FBSM with or without 2 ng/ml active human TGF-β1 for 24 h. **b-c**. Total RNA from treated (TGF-β1) or untreated (Ctrl) cells was isolated and the relative expression of uPAR mRNA (B) or uPA mRNA (C) was analysed using RT-qPCR. The error bars show the +SD. *N* = 3. **d**. Cells cultured in SFM or FBSM and stimulated with 2 ng/ml TGF-β1 for 24 h as indicated and PAI-1 protein levels were analysed. Controls received either no additives (−) or the TGF-β1 buffer (0). Positive control: recombinant mouse PAI-1 (rmPAI-1). **e**. AT84-uPAR cells cultured in either SFM or in FBSM. Cells were either unstimulated (−) or stimulated (+) with 2 ng/ml TGF-β1 and/or 10 μM of the TGF-β1-inhibitor SB431542 as indicated. **f**. AT84-uPAR cells were cultured for 24 h in FBSM and stimulated with 2 ng/ml TGF-β1 as indicated. **g**. Deglycosylated whole cell lysates from AT84-uPAR cells cultured in SFM stimulated with 2 ng/ml TGF-β1 +/− the inhibitor SB431542 as indicated were analysed for uPAR protein levels. **h**. Cells were cultured for 24- or 48 h in SFM, FBSM or TMEM. Cells were treated with 2 ng/ml TGF-β1 in 10% FBS as indicated. The LRP1 protein is indicated. Arrowhead shows an unknown band

We recently reported that medium containing soluble factors from the tumour microenvironment (TMEM) of the neoplastic leiomyoma tissue were the main regulators of both glycosylation and cleavage of uPAR [35], where TGF-β1 was found to be a major constituent of the soluble TME fraction [50]. To analyse whether TGF-β1 was one of the regulators of the observed uPAR cleavage, AT84-uPAR cells were treated with 2 ng/ml active recombinant human TGF-β1 for 24 h in FBSM. TGF-β1 induced a clear shift towards more full-length uPAR (Fig. 2a), confirming a role of TGF-β1 in regulating uPAR cleavage. In contrast to previous reports [51, 52], this shift was not due to a large increase in uPAR mRNA expression (Fig. 2b; not significant), nor as a result of decreased mRNA expression of the main uPAR cleaver uPA (Fig. 2c; not significant).

uPA is inhibited by PAI-1, a well-known down-stream target of TGF-β1 [53–57]. We therefore wanted to test whether TGF-β1 could induce PAI-1 expression in the AT84 cells, and thus inhibit uPA activity and uPAR cleavage. The AT84-EV and AT84-uPAR cells were cultured with SFM +/− TGF-β1 or FBSM +/− TGF-β1. As a control, the cells were also cultured in SFM or FBSM containing the buffer used for dissolving TGF-β1. A well without cells, but with FBSM +/− TGF-β1, was included as a negative control. After 24 h of incubation, the medium was analysed for the presence of PAI-1 by Western blotting (Fig. 2d). As expected, TGF-β1 induced PAI-1 expression in the AT84-EV and AT84-uPAR cells in both SFM and FBSM. There was also a slight difference in the MW of the PAI-1 band when the cells were grown in SFM compared to when FBS was present. Taken together, this shows that TGF-β1 is able to induce PAI-1 expression in both AT84-EV and AT84-uPAR regardless of the presence of FBS.

In order to verify that this effect was indeed mediated by TGF-β1, treatment with the specific TGF-β1 inhibitor SB431542 completely abolished the TGF-β1-induced PAI-1 expression in the AT84-uPAR (Fig. 2e). To test whether TGF-β1 had in fact reduced uPAR cleavage through increased PAI-1 expression, an anti-PAI-1 antibody was added to AT84-uPAR cells stimulated with TGF-β1 (Fig. 2f). The blocking antibody resulted in a shift towards more cleaved uPAR relative to full-length uPAR. To further verify these results, we could also show that inhibiting TGF-β1 signalling using SB431542 resulted in more uPAR cleavage in AT84-uPAR cells compared to controls stimulated with only TGF-β1 in SFM (Fig. 2g).

Low-density lipoprotein receptor-related protein 1 (LRP1) is an endocytic receptor for uPAR [58]. As reported by others, once PAI-1 binds uPAR-bound uPA, the uPAR/uPA/PAI-1 complex is endocytosed via LRP1, and full-length uPAR is rapidly recycled back to the cell surface [58, 59]. Different LRP1 levels might influence the amount of uPAR, uPA and PAI-1, which again could influence uPAR cleavage. Thus, we tested whether the different growth media would influence the level of LRP1. AT84-uPAR cells were cultured in either SFM, FBSM, TMEM or TGF-β1 and lysates were analysed by Western blot for LRP1 (Fig. 2h). Equal amounts of LRP1 could be detected in lysates from AT84-uPAR cells regardless of growth medium and stimulation with TGF-β1. The anti-LRP antibody recognizes an additional protein of a lower MW in samples containing FBS (black arrowhead). The identity of this protein is unknown. Thus, the difference in cleavage of uPAR is not due to different levels of LRP1.

Taken together, these results show that TGF-β1 can increase PAI-1 expression in the AT84 cells and thus reduce cleavage of uPAR, most likely via inhibition and internalization of uPA.

### Inhibition of uPA-activity reduces cell migration and invasion in a uPAR-dependent manner

**Fig. 3:**
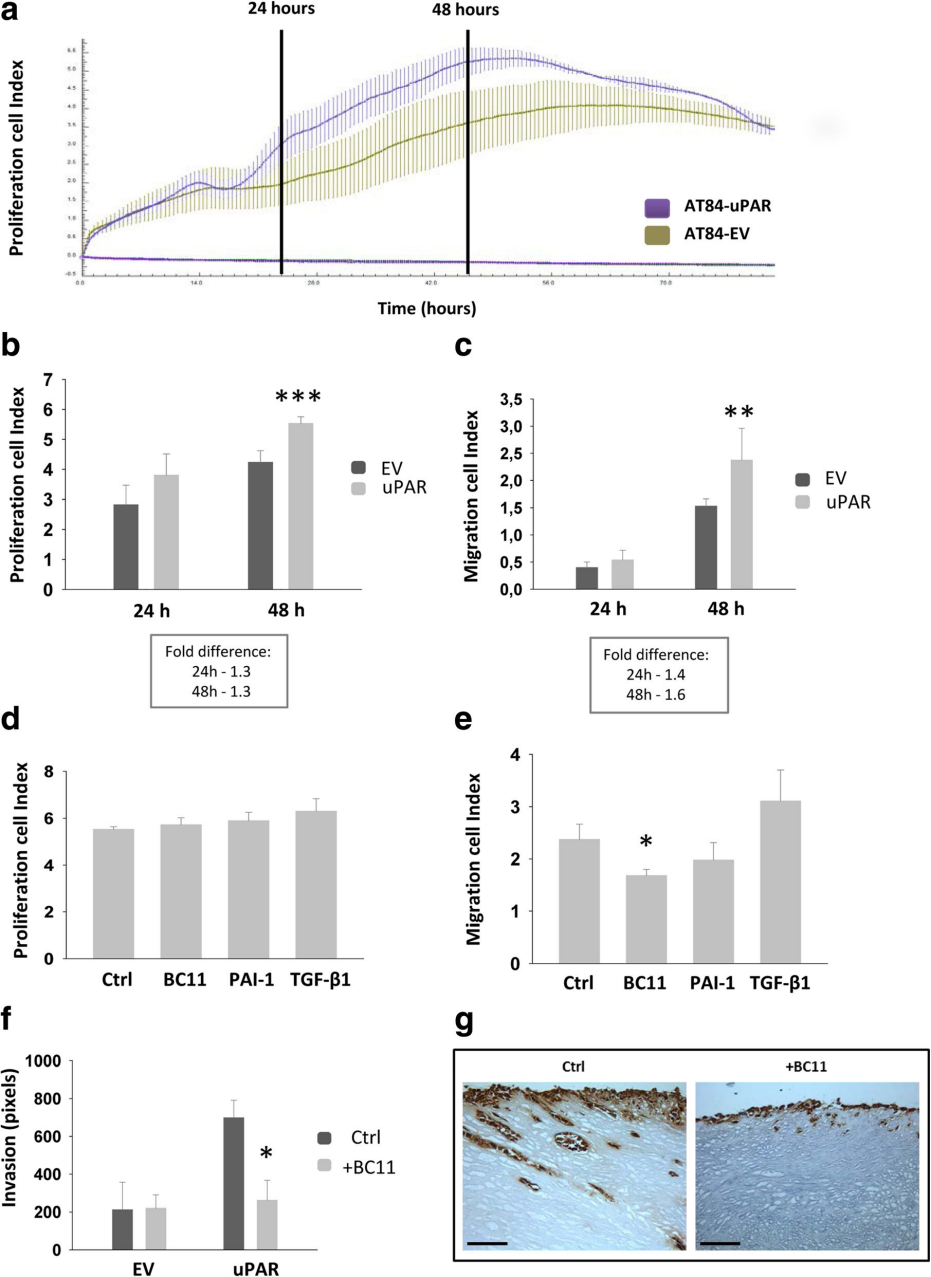
Inhibition of uPA-activity reduces migration and invasion in an uPAR-dependent manner. **a-e**. Real-time cell analysis of proliferation and migration of the AT84-EV and AT84-uPAR cells using the xCELLigence system. *N* = 3, where each experiment had two technical replicates. The y-axis shows the arbitrary “cell index” value based on cell adhesion induced impedance. Error bars show the ±SD. Student T-test; * *p* < 0.05, ** *p* < 0.005, *** *p* < 0.001. Fold difference was calculated using the mean values. **a**. One representative proliferation experiment. The standard deviation is based on the two technical replicates. The two vertical black lines show the 24- and 48 h points. Purple line = AT84-uPAR, green line = AT84-EV. Flat purple line = negative control; no cells added. **b**. Cell index values collected from three separate proliferation experiments at 24- and 48 h. **c**. Cell index values collected from three separate migration experiments at 24- and 48 h. **d**. Proliferation of AT84-uPAR cells treated with 10 μM BC11, 10 nM rmPAI-1 or 2 ng/ml TGF-β1 for 48 h. Controls received no additives. **e**. Migration of AT84-uPAR cells treated with 10 μM BC11, 10 nM rmPAI-1 or 2 ng/ml TGF-β1 for 48 h. Controls received no additives. **f**. AT84-EV and AT84-uPAR cells invaded the tissue of the leiomyoma invasion model for 7 days with or without the uPA-inhibitor BC11 hydrobromide present (+BC11). Controls received no additives (Ctrl), *N* = 3. Invasion depth was determined using at least 6 measurements per tissue section. The bars show the mean values of the three discs and the error bars show the ±SEM (*N* = 3). **g**. Representative tissue sections of invading AT84-uPAR cells were immunohistochemically stained for uPAR (right panels). Positive uPAR staining is seen as brown colour, counterstained with haematoxylin. Images were recorded at 20 x magnification

In order to analyse the functional effects of high levels of uPAR in AT84 cells, a real-time cell proliferation assay was performed in FBSM (Fig. 3a). Fold differences in cell index values from 24- and 48 h from three individual experiments were compared (Fig. 3b). AT84-uPAR cells gave a cell index value that was higher than that obtained from AT84-EV cells, suggesting a slightly higher proliferative rate. At 24- and 48 h, the fold difference in proliferation between the two cell lines was constant at 1.3 and statistically significant (*p* < 0.001) at 48 h (Fig. 3b).

Both full-length and cleaved human uPAR have been reported to play a role during cancer cell migration [28]. Hence, the AT84-EV and AT84-uPAR cells were analysed for their ability to migrate using FBSM as an attractant in a real-time cell analysis of migration. Under these conditions, most of the uPAR would be cleaved as shown in Fig. 1f. AT84-uPAR cells displayed a 1.4 fold increase in migration compared to the AT84-EV cells at 24 h, and 1.6 fold at 48 h (*p* < 0.005)(Fig. 3c).

To analyse whether inhibiting uPA activity, and thus also uPAR cleavage, would influence cell migration and proliferation, AT84-uPAR cells were analysed for proliferation and migration in the presence of the uPA inhibitors PAI-1 or BC11, and TGF-β1. None of these had any effect on proliferation (Fig. 3d), but interestingly inhibiting uPA activity using BC11 significantly reduced migration (Fig. 3e, p=0.008). Although not statistically significant, there was also a tendency towards reduction of migration when PAI-1 was present. Not surprisingly, as TGF-β1 has multiple effects on cells, a slight increase in migration was induced, though not statistically significant.

Analysis of invasion using the leiomyoma invasion model [60] showed that AT84-uPAR cells invaded more deeply than AT84-EV cells (Fig. 3f). Furthermore, inhibition of uPA activity using the uPA inhibitor BC11 significantly reduced tissue invasion by AT84-uPAR cells (Fig. 3f and g). No reduction of invasion was observed in the AT84-EV cells, even though both expression and activation of uPA in these cells was equal to that of the AT84-uPAR cells (see Fig. 1c and d). Taken together, these results indicate that uPAR and uPA together induce migration and invasion of the AT84 OSCC cells, and inhibition of uPA activity reduces both migration and invasion in an uPAR-dependent manner.

### Soluble uPAR shed from mouse OSCC cells induce migration in a paracrine manner

**Fig. 4:**
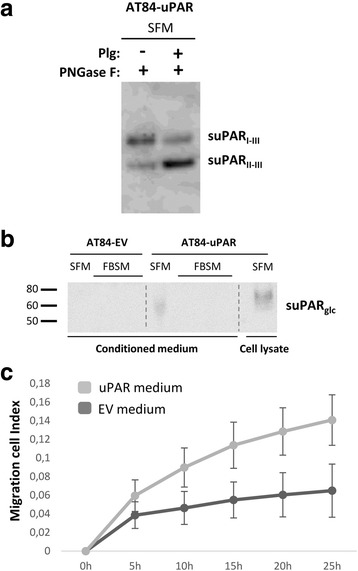
Soluble uPAR shed from mouse OSCC cells induce migration. **a-b**. Western blot analysis of uPAR protein levels in concentrated conditioned medium from AT84-EV and AT84-uPAR cells. **a**. Conditioned medium from cells cultured in SFM or SFM containing 10 nM plasminogen (plg) as indicated. The conditioned medium was concentrated from 2 ml to 110 μl for both samples, and deglycosylated using PNGase F. **b**. Conditioned medium from cells cultured in SFM or in FBSM was concentrated from 2 ml to 350 μl for each sample. A whole cell lysate sample of AT84-uPAR cells was used as a positive control. The two indicated FBSM samples are replicates. Glycosylated suPAR is indicated as suPAR_glc_. **c**. Non-concentrated conditioned medium from AT84-EV (EV medium) and AT84-uPAR cells (uPAR-medium) was used as an attractant for the AT84-EV cells in a real-time cell migration analysis using the xCELLigence system. *N* = 3, where each experiment had two technical replicates, and the error bars represent the standard error of mean (±SEM) based on three separate experiments

When human uPAR is cleaved between domains I and II a chemotactic peptide sequence (SRSRY) that remains on uPAR (II-III) may be revealed [61]. However, less is known about whether the equivalent amino acid sequence in mouse uPAR, PQGRY [17], can stimulate migration. Both full-length and cleaved human uPAR can be shed from the cell surface and have been found as soluble forms, termed suPAR and suPAR (II-III), respectively [28]. To analyse whether suPAR and suPAR (II-III) were detectable in the conditioned medium from AT84-uPAR cells, the serum-free conditioned medium was concentrated, deglycosylated and analysed by Western blotting (Fig. 4a). Both forms of suPAR were found present in the concentrated conditioned medium, however most of suPAR was full-length. Interestingly, when plasminogen was added to cultured cells, this resulted in markedly more suPAR (II-III) in the conditioned medium, reflecting the ratio observed on the cell surface as shown in Fig. 1h and further emphasizing the role of plasmin in regulation of uPAR cleavage. SuPAR could not be detected in the concentrated conditioned medium from AT84-EV cells, nor in AT84-uPAR concentrated conditioned FBSM (Fig. 4b), as expected due to serum-induced inhibition of phospholipases [62, 63]. To test whether mouse suPAR could function as a chemoattractant as previously reported for human uPAR [64], the conditioned SFM from AT84-EV and AT84-uPAR cells was harvested and used as an attractant in a migration assay using the AT84-EV cells (Fig. 4c). The AT84-EV cells migrated to a significantly larger extent towards the conditioned medium from the AT84-uPAR cells compared to the medium from the AT84-EV cells, suggesting that also mouse suPAR/suPAR (II-III) may enhance migration of the AT84 OSCC cells in a paracrine manner.

## Discussion

We previously found that uPAR expression was up-regulated at the tumour-stroma border in a mouse model for OSCC [35]. Soluble factors derived from the tumour microenvironment were shown to contribute to the increased expression of uPAR, but were also implicated in cleavage of the receptor. Cleavage of uPAR may regulate its many functions related to migration and invasion [28]. In the current study we aimed at analysing the effect of TGF-β1 and uPA on uPAR cleavage and the potential implications for cell proliferation, migration and invasion.

Overexpressing uPAR in the AT84 cells was shown to slightly increase the cells ability to migrate in 2D–culture (Fig. 3c). Moreover, inhibition of uPA activity with the subsequent shift towards more full-length uPAR, reduced both migration and invasion in a uPAR-dependent manner (Fig. 3e and f), suggesting functional relevance of the cleavage status of uPAR. It should however be kept in mind that uPA is one of the main activators of the broad-spectrum protease plasmin, which can result in further activation of other proteases such as MMPs [8]. Hence, by inhibiting uPA, the end result is not simply a reduction in uPAR cleavage. Interestingly though, inhibition of uPA activity in AT84-EV cells, expressing equal levels of active uPA, had no effect on invasion (Fig. 3f). This indicates that uPA exerts its invasion- and migration-promoting effects in concert with uPAR. It has been shown that membrane bound uPAR (II-III) can, at least in some types of human cells, stimulate migration through exposure of the SRSRY-peptide located between domain I and II of human uPAR [61]. uPAR (II-III) interacts with formyl-peptide receptors (FPRs), a type of G-protein coupled receptor (GPCR), via the SRSRY peptide, resulting in increased migration [27, 64–67]. In mouse uPAR, the corresponding amino acid sequence is PQGRY [17], and less is known about whether this mouse uPAR peptide encompasses similar functionality. The closely related rat uPAR peptide, PRGRY, has been reported to induce chemotaxis and cytoskeletal rearrangement [68]. The amino acids R and Y in the peptides are highly conserved and it has been shown that Y92 in human uPAR is the essential amino acid in triggering activation of the GPCR downstream effectors p56/59(hck) [27, 69]. Both GPI-anchored human uPAR (II-III) and suPAR (II-III) are reported to induce migration [28]. Both of these fractions could potentially contribute to the migration and invasion seen in Fig. 3. However we could only detect suPAR in the serum free conditioned medium and not the FBSM (Fig. 4b). Hence, the uPAR-induced migration seen in Fig. 3, which was performed in the presence of FBS, is most likely induced through the membrane bound form.

We also tested whether media containing uPAR shed from the cell surface could induce migration. uPA-induced uPAR cleavage is performed most efficiently when uPAR is GPI-anchored to the cell surface [20, 23], hence uPAR is probably cleaved before it is shed. In support of this, the levels of suPAR (II-III) and suPAR (I-III) reflected the levels observed on the cell surface (Figs. 4a and 1h). Gilder et al. recently showed that suPAR may function in a paracrine manner, enhancing migration and invasion of glioblastoma cells [70]. Furthermore, the levels of suPAR, suPAR (II-III) and suPAR (I) in biological fluids have all been linked to poor prognosis of several different cancer types [32, 71, 72]. To the best of our knowledge it has not previously been shown that mouse suPAR can induce chemotaxis. An analysis of the serum free conditioned medium revealed that the majority of the shed uPAR was full-length (Fig. 4a). Using the conditioned medium as an attractant during cell migration clearly induced AT84 cell migration compared to medium without suPAR (Fig. 4c). Hoyer-Hansen et al. used an SRSRY-recognising antibody and showed that the SRSRY-peptide could not be detected when uPAR was solubilised in its full-length version [20]. Hence, the chemotaxis inducing SRSRY-sequence of human uPAR is most probably not exposed on soluble full-length uPAR. Though the amount of suPAR (II-III) in the conditioned medium used here was very low compared to the full-length uPAR, only very low molar concentrations of the peptide are needed to induce migration [64]. As previously mentioned, mouse uPAR does not contain this exact SRSRY-sequence, and whether it is the PQGRY-sequence of mouse uPAR that induces the migration that we observe, or whether it is induced through another pathway is an interesting topic for future investigations. Nevertheless, the finding that media containing mouse suPAR has chemotaxic capabilities shows that expression of mouse uPAR in AT84-cells is a good model for further investigations of uPAR cleavage in vivo as it resembles human uPAR also in this respect.

We previously found that soluble factors from the TME of the leiomyoma tissue up-regulated uPAR expression and glycosylation and influenced uPAR cleavage [35]. Through analysis of the soluble TME fraction, TGF-β1 was found to be a major constituent [50]. We could now show that TGF-β1 is involved in regulating uPAR cleavage through stimulated PAI-1 secretion (Fig. 2). TGF-β signalling is either transmitted through SMADs, known as the canonical pathway, or through SMAD-independent (non-canonical) pathways, including tumour-necrosis factor (TNF) receptor-associated factor 4 (TRAF4) and TRAF6, p38 mitogen-activated protein kinase (p38 MAPK), RHO, AKT and ERK. Complicating this is the possibility that TGF-β signalling may be perturbed by other interacting pathways, epigenetic mechanisms, microRNAs and by corepressors [73, 74]. Interestingly, AT84 cells stimulated with TGF-β1 showed no change in proliferation at 48 h, but clearly increased PAI-1 expression. PAI-1 expression could be inhibited by SB431542, an inhibitor known to specifically inhibit the TβR-I proteins activin receptor-like kinases (ALK) -4, −5 and −7 [75], showing that the TGF-β1 receptor functions are intact. SMADs −2 and −3 are substrates for ALK4, −5 and −7, further transmitting the signal to the nucleus and initiating target gene transcription [75]. Why AT84-uPAR cell proliferation was unaffected by TGF-β1 stimulation even though the TGF-β1 receptor functions normally is an interesting target for future studies. Several different cell types in the TME secrete TGF-β1, which also functions as one of the major activators of fibroblasts, turning the fibroblasts into CAFs/myofibroblasts [76, 77]. Once activated, CAFs can furthermore secrete elevated levels of TGF-β1 [38, 78], which can be deposited in the TME. This could lead to increased secretion of PAI-1 from tumour cells, in turn inhibiting uPA activity and reducing uPAR cleavage. The end result being more full-length uPAR compared to cleaved uPAR. A reduction in uPA activity would most likely also reduce activation of plasmin and thus the overall proteolytic activity, contradicting the notion that TGF-β1 drives cancer progression. However, the TME is dynamic and stromal cells can be unequally distributed [79]. Furthermore, full-length uPAR interacts with a different subset of cell surface receptors compared to cleaved uPAR, enabling cancer cells to switch binding partners, modulate receptor functions, adhesion, migration and proteolytic activity in relation to TGF-β1 status [26, 27]. In oral cancer, α-SMA positive myofibroblasts are present to a greater extent in more invasive OSCC than in low-invading OSCC, and completely absent in normal tissue [80]. Similarly, TGF-β1 was not found expressed in normal oral mucosa, but increased expression was seen in oral leukoplakia and OSCC, including its reactive stroma [12, 13]. Interestingly, our previous findings show that up-regulated expression of PAI-1 (a target gene for TGF-β1) and uPAR in early stage OSCC correlated with poor disease specific survival [11]. Hence, further studies on the role of these components in uPAR cleavage are warranted in order to elucidate their role in OSCC progression.

## Conclusions

Taken together, these results demonstrate that OSCC cellular behaviour may be regulated not only through the levels of uPAR, but also through the balance between full-length uPAR and uPAR (II-III) present on cancer cells, and that TGF-β1 may control these events through the regulation of PAI-1 expression and subsequent uPA inhibition.

## Abbreviations

ATF: Amino terminal fragment
CAF: Cancer-associated fibroblast
EV: Empty vector
FBSM: Foetal bovine serum containing medium
gelzym: Gelatin zymography
LRP1: Low-density lipoprotein receptor-related protein-1
MMP: Matrix metalloprotease
OSCC: Oral squamous cell carcinoma
PAI: Plasminogen activator inhibitor
plgzym: Plasminogen zymography
PN-1: Protease nexin-1
SFM: Serum free medium
suPAR (I): Soluble domain I of uPAR
suPAR (II-III): Soluble uPAR lacking domain I
suPAR: Soluble uPAR
TAFI: Thrombin activatable fibrinolysis inhibitor
TGF-β1: Transforming growth factor – β1
TME: Tumour microenvironment
uPA: Urokinase plasminogen activator
uPAR (II-III): uPAR lacking domain I
uPAR: Urokinase plasminogen activator receptor
